# Incidence and mortality due to cervical cancer in 4 south European countries

**DOI:** 10.1016/j.pbj.0000000000000026

**Published:** 2018-08-03

**Authors:** Cristina Teixeira, Ana Afonso, Luciana Rodrigues, Muriela Madureira, António Nogueira

**Affiliations:** a Escola Superior de Saúde do Instituto Politécnico de Bragança, Bragança; b EPI-Unit, Instituto de Saúde Pública, Universidade do Porto, Porto; c Departamento de Ciências Veterinárias, CITAB, Universidade de Trás-os-Montes e Alto Douro (UTAD), Vila Real, Portugal.

**Keywords:** cervical cancer, incidence, mortality, Portugal, southern Europe

## Abstract

**Objective:**

To describe variation in incidence and mortality rates of cervical cancer (CC), between 4 Southern European countries that share similar cultural characteristics.

**Methods:**

Data on CC incidence and mortality reported in Portugal, Spain, Italy, and Greece for the year 2012 were obtained through the International Agency of Research on Cancer. Expected numbers of incident cases and deaths were obtained based on age-specific rates in European region. Standardized incidence rate (SIR) and standardized mortality rate (SMR) and respective 95% confidence interval (95% CI) were computed for each country by age group (15–39, 40–54, 55–64, and ≥65 years old).

**Results:**

The number of incident cases of and deaths due to CC observed in Greece, Italy, and Spain were significantly lower than expected, whichever the age group. In Portugal such pattern was, however, only found for incident cases among women aged up to 54 years. The number of incident cases observed in Portugal did not differ from that expected among women aged 55 to 64 (SIR = 90.8; 95% CI: 76.8–106.7) and aged 65 or more years (SIR = 110.0; 95% CI: 95.9–125.0). Also, the number of deaths observed in Portugal did not differ from that expected among women aged 15 to 39 (SMR = 70.0; 95% CI: 43.3–100.8), 40 to 54 (SMR = 93.6; 95% CI: 74.9–115.4), and 55 to 64 years (SMR = 93.6; 95% CI: 73.4–117.7) but was significantly higher than that expected among women aged 65 or more years (SMR = 126.7; 95% CI: 110.1–144.4).

**Conclusions:**

There is variability in CC incidence and mortality between 4 South European countries. To understand the reasons underlying such variability could improve approach to preventive care.

## Introduction

Cervical cancer (CC) is the fourth most common neoplasia and is one of the main causes of death due to cancer among European female population, particularly affecting young women.^1^

Cervical cytology (Papanicolaou test) screening has been used over the last decades for early detection of cervical lesions.^2,3^ The population-based screening programs, if well-organized, have been an effective measure in decreasing incidence and mortality due to CC.^4^

In these circumstances, the geographic variation for CC incidence and mortality reflects not only cultural differences but also differences in the way health systems provide screening programs for early detection and timely quality treatment.

The aim of this study is to assess variation in incidence and mortality due to CC across 4 South European countries that share similar cultural characteristics.

## Methods

Data on estimated CC incidence and mortality rates (per 100,000 women) for the year 2012 reported in Portugal, Italy, Spain, and Greece were obtained from International Agency for Research on Cancer through GLOBOCAN 2012.^1^ Observed incident cases and deaths for each country were estimated according to 4 age groups (15–39, 40–54, 55–64, and 65 years or more). Expected incident cases and deaths for each country and each age group were computed based on age-specific rates of incidence and mortality reported in European region.

Standardized incidence ratio (SIR) and standardized mortality ratio (SMR) and respective 95% confidence interval (95% CI) were computed by using the WinPepi software (available on: http://www.brixtonhealth.com/pepi4windows.html).

## Results

Regardless of age group, incidence and mortality rates were higher among Portuguese women. Whichever the country, women aged between 55 and 64 years old presented higher incidence rates varying from 11.5 in Greece to 21.5 in Portugal but mortality rates were higher among older women varying between 8.1 in Italy to 18.2 in Portugal (Table 1).

**Table 1:**
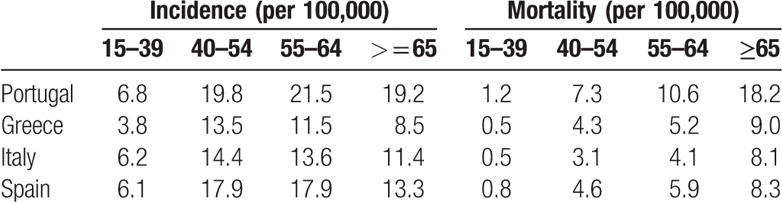
Incidence and mortality rates by country

**Figure 1:**
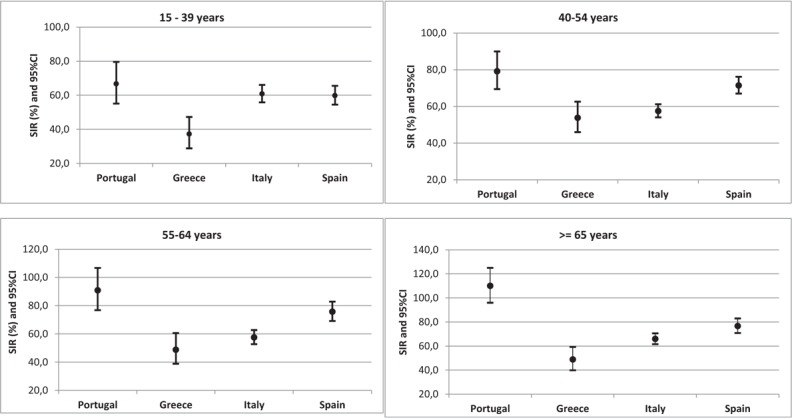
SIR for cervical cancer (CC) by age group in 4 South European countries (reference population: European region). CI = confidence interval; SIR = standardized incidence ratio.

Greece, Italy, and Spain showed observed incident cases significantly lower than expected for all age groups. In Portugal such pattern was, however, observed only among younger women in whom SIR was 66.7 (95% CI: 55.1; 79.6) for women aged 15 to 39 years and 79.2 (95% CI: 69.4; 90.0) among those aged 40 to 54 years. For older women the observed incident cases in Portugal did not differ from the expected; SIR values were 90.8 (95% CI: 76.8; 106.7) and 110.0 (95% CI: 95.9; 125.0) for age groups 55 to 64 and 65 or more years, respectively (Fig. 1).

**Figure 2:**
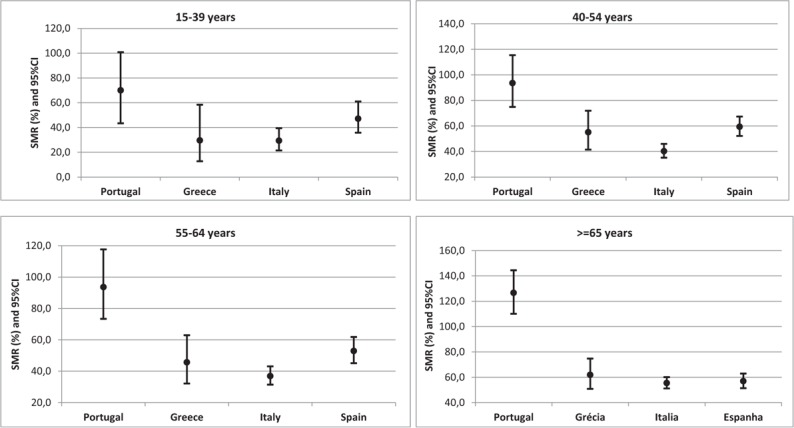
SMR for cervical cancer (CC) by age group in 4 South European countries (reference population: European region). CI = confidence interval; SMR = standardized mortality ratio.

Observed deaths due to CC in Portugal were significantly higher than expected for women aged 65 or more years who presented SMR value of 126.7 (95% CI: 110.1; 144.4) and similar to the expected for other age groups with SMR values of 70.0 (95% CI: 43.3; 100.8), 93.6 (95% CI: 74.9; 115.4) and 93.6 (95% CI: 73.4; 117.7) among women aged 15 to 39, 40 to 54 and 55 to 64, respectively. Instead the observed number of deaths in Greece, Italy, and Spain were significantly lower than expected (Fig. 2).

## Discussion

The results showed geographic variability in CC incidence and mortality between 4 South European countries that share similar cultural aspects. Although CC rates observed in Greece, Italy, and Spain were lower than the expected, rates observed in Portugal were similar to or higher than the expected. Portugal is the country with higher CC rates for 2012 which deserves particular attention.

The data presented in the International Agency of Research on Cancer database are the best available for each country worldwide. Caution must, however, be exercised when interpreting this information because the accuracy of estimates depends upon the quality and on the coverage of available data in each country.^1^

Human papillomavirus (HPV) infection, easily transmitted by sexual contact, causes almost all cases of CC.^2,5,6^ Geographic variability in CC rates has been partially explained by differences in prevalence of infection between populations.^7^

HPV vaccine is a safe measure that confers long-term protection against infection.^2,5,8^ Portugal, Spain, Greece, and Italy implemented publicly funded HPV immunization programs <10 years ago (2007/2008) and these programs target only preadolescent or adolescent girls or young women.^8^ Thus, there is a large group of women for whom the decrease of CC rates depends on the adherence to cervical screening for early detection of intraepithelial lesions.

Cervical cytology (Papanicolaou test) screening allows diagnosis of premalignant lesions, which can be treated to prevent invasive CC^2,3^ and the CC diagnosis at an earlier stage allowing more effective treatment with a consistent benefit in decreasing mortality.^2,3,9^ Therefore, well-organized population-based cervical screening programs have a strong impact in decreasing CC rates.^4,9^

From all countries here analyzed, only Italy and Portugal reported population-based cervical screening programs which began in 1989/1990. These programs are, however, still in the process of rolling out with no total coverage of population. The proportion of target population invited for cervical screening in 2013 was almost 70% in Italy but <20% in Portugal,^4^ which could partially explain the variability observed between Portugal and Italy in regards to CC rates.

Although Portugal reports population-based cervical screening programs, SIR and SMR values in Portugal are similar to or higher than the values observed in Greece and Spain where there are no population-based cervical screening programs, only opportunistic screening.^4^ This means that delivery of cervical screening in the community might not be enough in preventing CC. Efforts in community interventions and health promotion aiming to increase knowledge about CC prevention and treatment could make the difference in decreasing the risk of HPV infection and in increasing uptake of available screening programs.^6,9–11^ Strategies to increase adherence to cervical screening programs could play an important role, particularly among Portuguese older women who reported higher prevalence of nonusers or under-users of cervical screening.^12^

Further research could give insights about factors underlying the unfavorable CC incidence and mortality rates observed in Portugal. The knowledge about such factors will make possible to adopt preventive measures in decreasing CC incidence and mortality.

## Acknowledgments

None.

## Authors contributions

CT and AN conceived the study. AA, LR, and MM collected data. CT, AA, LR, and MM analyzed data. All authors contributed to the interpretation of results, commented on drafts, and approved the final version.

## Conflicts of interest

The authors declare no conflicts of interest.
